# Six-Month Mortality among HIV-Infected Adults Presenting for Antiretroviral Therapy with Unexplained Weight Loss, Chronic Fever or Chronic Diarrhea in Malawi

**DOI:** 10.1371/journal.pone.0048856

**Published:** 2012-11-19

**Authors:** Monique van Lettow, Ann Åkesson, Alexandra L. C. Martiniuk, Andrew Ramsay, Adrienne K. Chan, Suzanne T. Anderson, Anthony D. Harries, Elizabeth Corbett, Robert S. Heyderman, Rony Zachariah, Richard A. Bedell

**Affiliations:** 1 Dignitas International, Zomba, Malawi; 2 University of Toronto, Toronto, Canada; 3 Médecins Sans Frontières, Thyolo, Malawi; 4 George Institute for Global Health and the University of Sydney, Sydney, Australia; 5 World Health Organization, Tropical Disease Research Programme, Geneva, Switzerland; 6 School of Medicine, University of St Andrews, Fife, Scotland; 7 Malawi-Liverpool-Wellcome Trust Clinical Research Programme, University of Malawi College of Medicine, Blantyre, Malawi; 8 Brighton and Sussex Medical School, Falmer, United Kingdom; 9 International Union Against Tuberculosis and Lung Disease, Paris, France; 10 London School of Hygiene and Tropical Medicine, London, United Kingdom; 11 Médecins Sans Frontières, Operational Research Unit, Brussels Operational Centre, Luxembourg, Luxembourg; 12 Division of Global Health, University of British Columbia, Vancouver, Canada; University of Toronto, Canada

## Abstract

**Background:**

In sub-Saharan Africa, early mortality is high following initiation of antiretroviral therapy (ART). We investigated 6-month outcomes and factors associated with mortality in HIV-infected adults being assessed for ART initiation and presenting with weight loss, chronic fever or diarrhea, and with negative TB sputum microscopy.

**Methods:**

A prospective cohort study was conducted in Malawi, investigating mortality in relation to ART uptake, microbiological findings and treatment of opportunistic infection (OIs), 6 months after meeting ART eligibility criteria.

**Results:**

Of 469 consecutive adults eligible for ART, 74(16%) died within 6 months of enrolment, at a median of 41 days (IQR 20–81). 370(79%) started ART at a median time of 18 days (IQR 7–40) after enrolment. Six-month case-fatality rates were higher in patients with OIs; 25/121(21%) in confirmed/clinical TB and 10/50(20%) with blood stream infection (BSI) compared to 41/308(13%) in patients with no infection identified. Median TB treatment start was 27 days (IQR 17–65) after enrolment and mortality [8 deaths (44%)] was significantly higher among 18 culture-positive patients with delayed TB diagnosis compared to patients diagnosed clinically and treated promptly with subsequent culture confirmation [6/34 (18%);*p* = 0.04]. Adjusted multivariable analysis, excluding deaths in the first 21 days, showed weight loss >10%, low CD4 count, severe anemia, laboratory-only TB diagnosis, and not initiating ART to be independently associated with increased risk of death.

**Conclusions:**

Mortality remains high among chronically ill patients eligible for ART. Prompt initiation of ART is vital: more than half of deaths were among patients who never started ART. Diagnostic and treatment delay for TB was strongly associated with risk of death. More than half of deaths occurred without identification of a specific infection. ART programmes need access to rapid point-of-care-diagnostic tools for OIs. The role of early empiric OI treatment in this population requires further evaluation in clinical trials.

## Introduction

Mortality among patients initiating antiretroviral therapy (ART) is high in the first months of treatment [1]–[3], especially in low income countries. Between 8% and 26% of patients die in the first year of ART [1], [2]. Immunological and virological responses to ART are similar in low and high income countries [1].

Causes of early death among patients on ART have been well documented and include: tuberculosis (TB), sepsis, cryptococcal meningitis (CM), malignancies, chronic diarrhea and wasting syndrome [2]–[4]. Established risk factors include advanced immunosuppression, WHO Clinical Stage 3 and 4, wasting, anemia and payment for ART [1], [3], [5], [6]. Conversely, prompt initiation of ART can reduce mortality [2], [3], [5].

Deaths occurring between enrolment and actual initiation of ART are less often reported, but are high. In two South African cohorts, the numbers of deaths during this interval exceeded early deaths after ART initiation [5], [7]. Pulmonary and CNS infections, notably TB, are the most common causes of serious opportunistic infections (OIs) [8]–[13] in HIV-infected Africans. The etiology of most fatal infections is determined only at autopsy [14].

Two previous studies in Malawi have shown that about half of patients classified as WHO Stage 3 had unexplained weight loss, chronic fever or chronic diarrhea [15]. Adults with these symptoms have inferior early ART treatment outcomes compared to those starting ART with other stage 3 diagnoses [16], most likely relating at least in part to a high burden of active TB and/or bloodstream infections (BSIs) that are difficult to diagnose in resource-limited settings [17]–[24]. Once stabilised on treatment, ART substantially reduces the risk of OIs, including TB. As well, ART subsequently reduces all-cause mortality [25].

By the end of 2009, 271,105 HIV-infected individuals had been registered on the national ART programme in Malawi and Cotrimoxazole Preventive Therapy (CPT) had been widely implemented [26]. In this context the aim of this study was to investigate mortality 6 months after the intention to initiate ART, in a cohort of ambulatory HIV-infected smear-negative Malawian adults investigated for TB and serious BSIs [27]. The specific objectives were to: i) describe 6-month outcomes in relation to uptake of ART and treatment of serious infections (including TB) ii) determine risk factors for death and iii) assess whether 6-month outcomes were affected by different diagnostic processes.

## Methods

### Ethics Statement

This study received prior ethical approval from the National Health Sciences Research Committee of Malawi and from the Ethics Advisory Group of the International Union Against Tuberculosis and Lung Disease. Both ethics committees approved the consent form and consent procedure. All study participants provided written informed consent.

### Study Design

A prospective cohort study, conducted at two hospitals in Malawi.

### Study Setting

The study was carried out in the ART outpatient clinics at Zomba Central Hospital and Thyolo District Hospital, serving 2 districts in the southern region of Malawi. Malawi has a high HIV co-infection rate of 60–70% among TB patients [28] and an adult HIV prevalence of approximately 12% [29]. The TB incidence in Malawi in 2010 was estimated at 219/100,000 general population [30]. It is the national policy in Malawi to prescribe CPT to all HIV-infected persons. Patient enrolment took place from February to December 2010 and outcome data were obtained for the period February 2010 to June 2011.

### Study Participants

Patients enrolled into the study were ambulatory HIV-infected adults (≥15 years of age) who were intended to start ART and presented with unexplained weight loss, chronic fever or chronic diarrhea, with negative expectorated sputum smear microscopy for TB [27]. Patients able to expectorate had sputum smear microscopy either prior to, or during the screening process, on three sputum specimens as per Malawi National TB Program guidelines.

### Study Interventions

The detailed methods, including laboratory methods and procedures used in this study (to identify TB and BSIs) have been described elsewhere [27]: in brief, on the day of enrolment all patients had a symptom screen, blood culture (including TB), cryptococcal antigen test (CrAg), a single induced sputum (IS) specimen for TB microscopy and solid culture, full blood count and CD4 lymphocyte count. Diagnostic test results were relayed by study personnel to treating clinicians for treatment decisions.

### Study Variables and Data Collection Instruments

Baseline characteristics of patients included: sex, age, weight loss>10%, chronic fever, chronic diarrhea, lymphadenopathy, oral candidiasis, Body Mass Index (BMI), CD4 count and hemoglobin (Hb). BMI, CD4 and Hb were categorized using conventional cut-off values [27]. Severe anemia was defined as Hb <80 g/L (F) and <90 g/L (M); moderate anemia as Hb 80–109 g/L (F) & 90–119 g/L (M); mild or no anemia as Hb ≥110 g/L (F) & ≥120 g/L (M). Diagnosed infections included: TB diagnosed on clinical/radiological grounds either at enrolment, or ≥21 days after enrolment, *M. tuberculosis* (MTB) confirmed through smear or culture, or a BSI confirmed through culture. The interval of 21 days from enrolment was chosen as a reasonable time for sub-clinical TB to be unmasked with reconstitution of the immune system and/or potentially more intensive clinical surveillance and care during early ART initiation.

### Sources of Cohort Data

Diagnosed infections, treatment uptake and timing, and 6-month outcomes were retrieved from patient files, TB registers and ART databases. In cases where 6-month outcomes were not reported, patients were traced to their homes. Information obtained through tracing included verification of uptake and timing of ART and treatment of BSIs in patients’ personal health books, and/or date of death.

### Data and Statistical Analysis

Baseline characteristics, diagnosed infections, uptake of treatment and timing of treatment uptake or death were described with proportions or medians (interquartile ranges [IQR]). Uptake of treatment included: ART among all, TB treatment among TB cases and antimicrobial treatment among BSI cases. Timing of treatment uptake was defined as the interval between enrolment and treatment start in days, where enrolment was the point at which a patient was determined to be eligible for ART initiation.

Comparisons between groups were made using chi-square tests; non-parametric independent sample median tests were used for variables with non-normal distribution. Multivariable logistic regression models were fitted with “death within 6 months of enrolment” as the outcome variable to determine risk factors for death. Covariates included baseline characteristics, diagnosis of an infection and uptake and timing of treatment. In order to ensure sufficient power, a minimum of 8 events per variable was defined *a priori* as the criterion required for inclusion of a predictor variable in the regression model [31]. All variables were simultaneously entered in the model as the first step and tested for removal one by one. The covariate “survived >21 days from enrolment/intention to start ART” was also included in the model to address the *immortal survival bias*, which is a selection bias occurring in the early observed effects where a cohort study design is being used to assess a treatment, as individuals need to survive first to receive the treatment [32]. The interval of 21 days was chosen, since in this study population all patients who survived this interval should have been started on ART when taking into account system delays (e.g. initiation of TB treatment for TB cases, arranging appointments, transport issues, pre-ART counseling, etc.). A significance level of 0.05 was set for all statistical testing.

Data and statistical analysis were conducted using IBM SPSS Statistics 19 (IBM, Armonk, NY, USA).

## Results

A total of 469 ART-eligible adults (58% female) were enrolled in the study from February to December 2010. Twelve of the 555 patients referred for assessment of eligibility in the study, declined to participate; 74 did not meet the inclusion criteria, as reported in detail, previously [27].

Of all 469 patients included in the study, 35 did not have a successful sputum induction; all of those had a negative TB blood culture result, and 4 had a clinical TB diagnosis. Out of these 35, at 6 months 20 were alive, 9 died and 6 were lost to follow-up.

**Figure 1:**
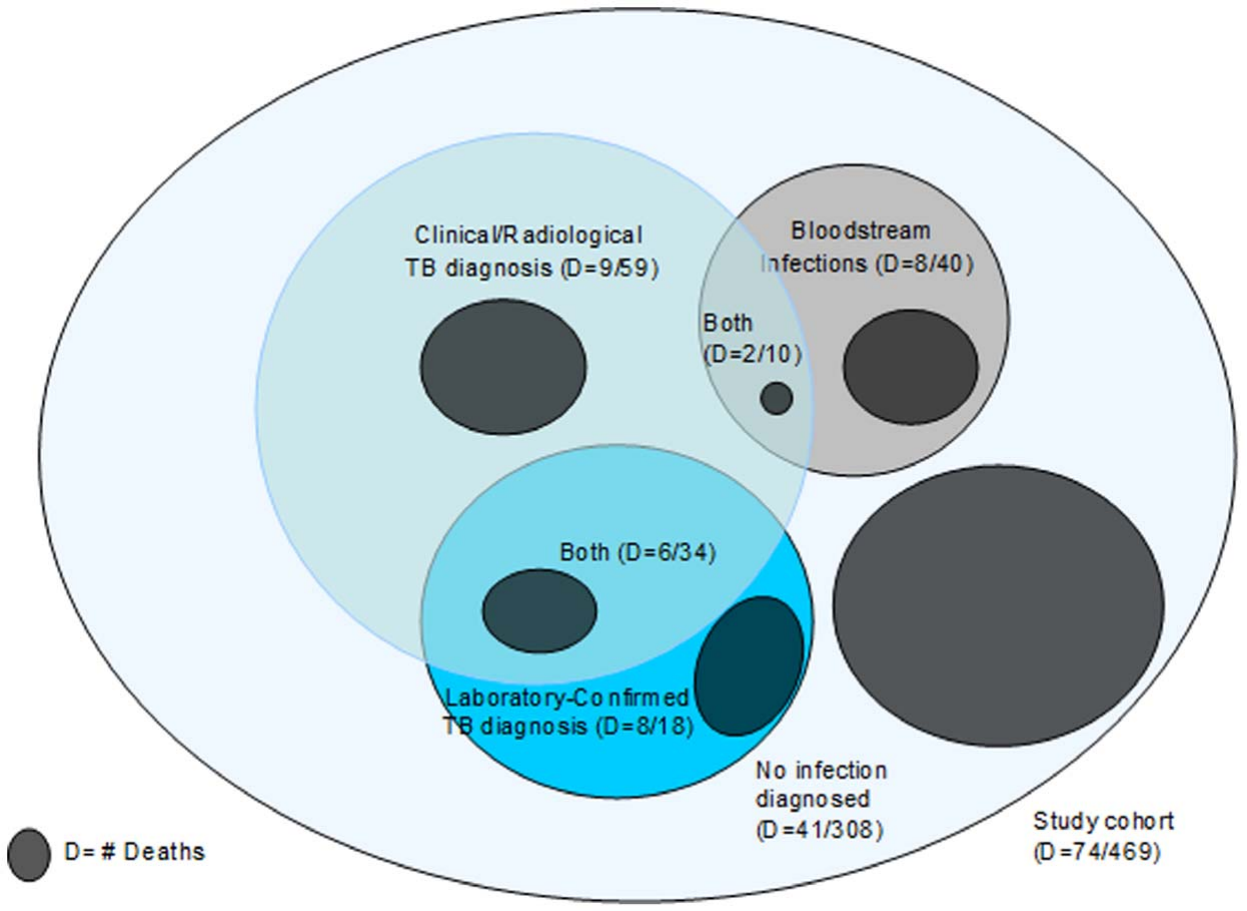
STUDY COHORT.

**Figure 1** illustrates the number and proportion of patients diagnosed with TB and/or BSIs and the number of individuals who died as part of the total study cohort.

Within 6 months, a total of 74 (16%) patients had died. Deaths occurred at a median of 41 days (IQR 20–81) from enrolment. Among all deaths, 20 (27%) were within 3 weeks (21 days) and 62 (84%) within the first 3 months after study enrolment. Among those who died within and after 21 days, the number and proportion diagnosed with TB was 3 (15%) and 11 (20%); and with BSIs, 6 (30%) and 4 (7%), respectively.

Cohort mortality at 6 months among patients with OIs was 423 deaths/1000 person-years, and 266 deaths/1000 person-years among patients with no infection identified.

**Table 1** shows 6-month outcomes by diagnosis and treatment uptake. A total of 103 (22%) patients were diagnosed with TB based on clinical/radiological grounds, including 34 (33%) where the decision to treat as TB preceded laboratory confirmation of TB. Ninety-two patients were clinically diagnosed with TB at enrolment and another 11 patients ≥21 days after enrolment. A further 18 (4%) MTB cases and 50 (11%) BSI cases were identified solely through additional laboratory investigations provided by the study. Mortality was highest among those who did not start ART and/or appropriate treatment for other infections, varying from 34% among those with no identified infection and not yet on ART to 100% among those with a laboratory confirmed BSI and not yet on ART nor anti-microbial treatment.

**Table 1:** Six-month outcomes by diagnosis and treatment uptake.

Diagnoses	Treatment Uptake	n	LTF/TO#	Alive	Mortality
**TB diagnosed on clinical grounds**		**103**	**23** (22%)	**63** (61%)	**17 (17%)**
TB diagnosed at enrolment	ART & TB Treatment	65	6	53	6 (9%)
	TB Treatment only	25	12	4	9 (36%)
	No ART & No TB Treatment	2	1	–	1 (50%)
TB diagnosed >20 days after enrolment	ART & TB Treatment	10	3	6	1 (10%)
	TB Treatment only	1	1	–	
**TB diagnosed through culture only**		**18**	**2** (11%)	**8** (44%)	**8 (44%)**
	ART & TB Treatment	11		7	4 (36%)
	TB Treatment only	3	1	1	1 (33%)
	No ART & No TB Treatment	4	1		3 (75%)
**BSI identified through culture** **		**50**	**6** (12%)	**34** (68%)	**10 (20%)**
	ART & Antimicrobial Treatment	40	4	34	2 (5%)
	ART only	4	2		2 (50%)
	No ART & No Antimicrobial Treatment	6			6 (100%)
**No infection identified**		**308**	**64** (21%)	**203** (66%)	**41 (13%)**
	ART	250	43	186	21 (8%)
	No ART	58	21	17	20 (34%)
**Totals** **		**469**	**94** (20%)	**301** (64%)	**74 (16%)**

# LTF/TO = Lost to follow up/Transferred Out.

* clinical = clinical & radiology.

** 10/50 BSI cases were also diagnosed with TB on clinical grounds and started on TB Treatment.

Among the total cohort, 370 (79%) started ART at a median of 18 days (IQR 7–40) from enrolment. Mortality among those who started ART and those who did not start ART was 34/370 (9%) and 40/99 (40%), or 184 and 808 deaths/1000 person-years, respectively (p = 0.001). In those who died, the median time to death was 71 days (IQR 40–89) for those who started ART versus 26 days (IQR 13–41) for those who had not started ART (p = 0.001).

**Table 2** shows the characteristics of the adults who died compared to those who survived 6 months from intention to start ART. A greater proportion of adults who died had baseline weight loss >10%, chronic diarrhea, severe anemia, a diagnosis of TB based solely on laboratory investigations, and not yet started ART (among all), not started TB treatment (among TB cases) or not started antimicrobial treatment (among BSI cases) compared to those who survived.

**Table 2:** Characteristics of ART-eligible adults who died within 6 months of intention to start ART versus those who are known to be alive.

		Adults who died within6 months n = 74	Adults who survived6 months n = 301	*P*-value	
Baseline characteristic	Sex, F	39 (53%)	177 (59%)	0.4	
	Median Age, in years (IQR)	35 (30–43)	36 (30–42)	0.9	
	Weight loss >10%	49 (66%)	143 (48%)	0.004	
	Chronic Fever	44 (59%)	183 (61%)	0.9	
	Chronic Diarrhea	34 (46%)	92 (31%)	0.01	
	Lymphadenopathy	27 (36%)	107 (36%)	0.9	
	Oral Candidiasis	24 (32%)	94 (31%)	0.9	
	BMI strata (kg/m2)*				
	Severe wasting; BMI<16.0	23 (31%)	74 (25%)	0.3	
	Moderate wasting; BMI 16.0–16.99	18 (24%)	59 (20%)		
	Mild or no wasting; BMI>17.0	33 (45%)	168 (56%)		
	CD4 strata (cells/µL)				
	CD4<50	22 (30%)	75 (25%)	0.7	
	CD4 50–199	35 (47%)	148 (49%)		
	CD4≥200	17 (23%)	78 (26%)		
	Hemoglobin strata (g/L)				
	Severe anemia	33 (45%)	66 (22%)	0.001	
	Moderate anemia	26 (35%)	145 (48%)		
	Mild anemia or no anemia	15 (20%)	90 (30%)		
TB and BSI’s	TB diagnosed on clinical grounds at enrolment	16 (22%)	57 (19%)	0.6	
	TB diagnosed on clinical grounds>20 days after enrolment	1 (1%)	6 (2%)	0.9	
	TB Laboratory confirmed only	8 (11%)	8 (3%)	0.005	
	BSI Laboratory confirmed	10 (14%)	34 (11%)	0.8	
	No infection	41 (55%)	203 (67%)	0.06	
Treatment	Started ART	34 (47%)	279 (93%)	0.001	
	Among those who started ART; Medianno of days from Enrolment to ART start (IQR)	17 (6–34)	20 (7–48)	0.7	
	Started TB Treatment (among TB cases)	21/25 (84%)	71/71 (100%)	0.004	
	Among those who started TB Treatment; Medianno of days from Enrolment to TB Treatment start (IQR)	7 (0–23)	0 (0–12)	0.1	
	Antimicrobial Treatment started (among BSI cases)**	2/10 (20%)	34/34 (100%)	0.001	

* Conform WHO strata for BMI grading of severity of malnutrition.

** Incomplete data on BSI treatment start dates.

**Tables 3** **and** **4** present the uptake and timing of treatment (ART, TB and antimicrobial treatment). In addition, these tables present 6-month mortality and timing of death by diagnosis of TB (based on clinical/radiological grounds), TB confirmed on culture, BSIs through laboratory investigations, or no infection identified.

Seventeen (17%) clinical/radiological TB cases died at a median of 50 days (IQR 26–81) from enrolment: 1 died before treatment commenced, 16 died on TB treatment (of whom 7 had also started ART). Mortality among the TB cases diagnosed solely on laboratory evidence (8/18) was significantly higher than that among TB cases where the decision to treat was based on clinical/radiological grounds (17/103), and among patients diagnosed clinically with subsequent culture confirmation (6/34): (44% vs. 17% and 18%, respectively; p = 0.04). While there was no difference in median time to ART initiation (p = 0.52), median TB treatment start was 27 days later (p = 0.01) for those individuals diagnosed solely using laboratory evidence compared to those diagnosed on clinical grounds. Among the total group of 50 patients with confirmed BSIs, 10 (20%) died (7 before receiving appropriate treatment), at a median of 20 days (IQR 12–41) from enrolment. Deaths among individuals with BSI’s included; 5/29 non-typhoid Salmonella (NTS), 1/8 C. neoformans, 1/5 E. coli, and 3/4 non-tuberculous mycobacteria (NTM). There were 10 BSI/TB (TB diagnosed on clinical/radiological grounded) co-infections, of whom 2 (with NTS) died.

**Table 3:** Case Management and Mortality of Individuals with a TB diagnosis.

	Proportion starting TB Treatment	Median (IQR) days to TB Treatment start	% starting ART	Median (IQR) days to ART start	Mortality 6 Months post-enrolment	Median (IQR) days enrolment-death
TB diagnosed on clinical grounds at enrolment	90/92	0 (0–7)	65 (71%)	23 (14–42)	16 (17%)	46 (22–84)
TB diagnosed on clinical grounds >20 days after enrolment	11/11	47 (32–84)	9* (82%)	10 (1–15)	1 (9%)	58
MTB Laboratory confirmed only	14/18	27 (17–65)	11 (61%)	9 (3–22)	8 (44%)	30 (26–85)

* TB diagnosed at a median of 29 days (IQR20–50) post ART initiation.

**Table 4:** Case Management and Mortality of Individuals with BSIs or No infection.

	Proportion starting Antimicrobial Treatment*	% started ART	Median (IQR) days to ART start	Mortality 6 Months post-enrolment	Median (IQR) days enrolment-death
BSI** Laboratory confirmed	40/50	44 (88%)	15 (6–34)	10 (20%)	20 (12–41)
No infection identified at enrolment	0/308***	250 (81%)	20 (7–46)	41 (13%)	42 (21–73)

* Incomplete data on BSI treatment start

** 10/50 were also diagnosed with TB on clinical grounds and started on TB Treatment.

*** no data available on possible empiric antimicrobial use among patients with no infection diagnosed.

Of the remaining 308 individuals with no identified infection, 81% started ART at a median of 20 days (IQR 7–46). Forty-one (13%) died at a median of 42 days (IQR 21–73) from enrolment of whom 21/41 died without having started ART at a median of 23 days (IQR 14–50) from enrolment.

In multivariable analysis (**Table 5**) baseline weight loss >10%, low CD4, severe anemia, being diagnosed with TB solely through laboratory investigations and not initiating ART, were independently associated with increased risk of death.

**Table 5:** Risk Factors associated with 6-month mortality among adults presenting for ART with unexplained weight loss, chronic fever or diarrhea, but negative TB sputum microscopy.

Risk Factor**	Adjusted OR* (95% CI)	*P*-value
Weight loss >10%	2.5 (1.2–5.1)	0.015
CD4 strata (cells/µL)		
CD4<50	3.6 (1.3–10.1)	0.016
CD4 50–199	3.4 (1.3–8.9)	0.014
CD4≥200	1	
Hemoglobin strata (g/L)		
^ #^Severe anemia	2.9 (1.2–6.9)	0.019
^ #^Moderate anemia	0.7 (0.3–1.7)	0.45
^ #^Mild anemia or no anemia	1	
MTB (Lab confirmed only)	4.3 (1.3–13.7)	0.001
No ART started	6.2 (2.7–14.1)	0.001

* Adjusted for baseline characteristics (sex, age, weight loss>10%, chronic fever, chronic diarrhea, lymphadenopathy, oral candidiasis, BMI, CD4, Hb, occurrence of an infection (TB diagnosed on clinical/radiological grounds at enrolment or >20days after enrolment, MTB confirmed through laboratory investigations, or a BSI confirmed through laboratory investigations) and uptake and timing of treatment (ART among all, TB treatment among TB cases, antimicrobial treatment (among BSI cases), in addition to survival of ≥21 days from enrolment.

# See methods for definitions.

** Only significant associations are reported in this table.

Patients needed to survive long enough to be able to start ART. To assess the impact of potential immortal survival bias, the association of not having started ART with mortality, with and without adjusting for survival ≥21 days, was adjusted OR (aOR) of 6.2 (95% CI 2.7–14.1) and aOR 10.5 (95% CI 5.1–21.9), respectively.

Induced sputum microscopy and culture identified 18 patients with MTB who did not already have a clinical diagnosis of TB, of whom 8 started TB treatment (7 also started ART) and are known to have survived 6 months. Among the 50 BSI cases, 34 started ART and antimicrobial treatment and are known to have survived.

## Discussion

This study provides knowledge from typical programme settings in Malawi and highlights that clinical decisions about antimicrobial treatment, especially TB treatment, are essential in order to optimize survival, even with high-quality laboratory support (where results are not available at point of care). Likewise, prompt ART initiation regardless of infection status appears to be associated with survival.

This study found that early mortality remains high among chronically ill HIV-infected patients with non-specific WHO Stage 3 or 4 criteria for ART initiation, even with the benefit of more intensive investigations for OIs than are usually afforded in resource limited settings.

More than half of all deaths were among the minority (21%) of patients who did not start ART, with the majority occurring within one month of enrollment. Weight loss >10%, low CD4 count (<200 and especially <50) and severe anemia were also significant risk factors for death.

Prompt initiation of ART in patients with suspected OIs is supported by a number of randomized controlled trials [8], [32]–[35] that have shown benefit to early ART initiation (within 2 weeks) in highly immunosuppressed patients, with the exception of those with CNS infections where the risks of fatal immune reconstitution inflammatory syndrome (IRIS) are high.

High mortality in this cohort of patients with advanced HIV further supports earlier initiation of ART before WHO stage 3 or 4 illness have developed [3]. Closer follow-up for individuals with HIV but not yet on ART, including regular CD4 count assessments, will be key to identifying relatively fit individuals in need of ART, and may be especially valuable in countries such as Malawi that have high rates of potentially fatal OIs but limited resources to diagnose OIs once established.

Initiation of ART and prompt TB treatment on clinical grounds before culture confirmation were associated with increased survival in this cohort. A recent study in South Africa found reduced mortality among inpatients managed according to the 2007 World Health Organization guidelines for management of severely ill HIV-infected patients (meeting expanded definitions for suspected smear-negative TB) compared to the “standard” management [33]. The 2007 recommendations stress the need to start TB treatment promptly without waiting for culture-confirmation.

In contrast, patients diagnosed solely through laboratory results initiated TB treatment with a median one-month delay and had substantially higher case-fatality rates. Our analysis suggests that enhanced laboratory identification using solid culture media and blood culture averted, at most, eight additional TB deaths implying that solid TB culture, even if routinely available, may offer only modest gains, a conclusion also reported from India [37]. Prompt initiation of TB treatment would be better supported through improved access to rapid point-of-care diagnostics for TB but in their absence empiric TB treatment warrants careful consideration, particularly among patients with the risk factors for mortality observed in this study.

We could not specifically evaluate the impact on mortality of time to initiation of ART after starting TB treatment. Recent literature and guidelines support starting ART as soon as possible or even at the same time as TB-treatment [33]–[36], [38] but this was not recommended in the Malawian national guidelines or made operational at a national level at the time of this study. Although 75% of TB patients started ART, more than one third of TB patients not initiating ART died, with half lost to follow-up, and only a minority known to be alive at 6 months. Advanced clinical disease, lack of coordination between ART and TB clinics, and patient reluctance to be treated for both diseasessimultaneously are likely contributing factors. Stigma and the fear of drug interactions may also have influenced ART uptake among these TB patients [28].

Among the small group of 10 patients with a diagnosis of TB made after initiation of ART, mortality was low. These patients started TB-treatment on average less than one month after ART initiation, suggesting that most were unmasked due to TB immune reconstitution inflammatory syndrome (IRIS). An observational study from South Africa found that initiation of ART prior to TB treatment was not associated with adverse outcomes [36]. This suggests that ART initiation should not be delayed if TB cannot be ruled out.

Our study found a high mortality rate (9/41, excluding CM) among patients with BSIs. NTS were especially common among our cohort of chronically ill HIV-patients. Salmonella species have also predominated in several other African studies, particularly in high HIV prevalence settings [9], [10]. In a cohort of patients with NTS infections in Malawi in the pre-ART era, 98% of patients tested were HIV-positive; the inpatient mortality rate was 47% and recurrence rate 43% for the survivors [39]. Among our patients, 21 of 29 patients with NTS survived, 3 were lost to follow up; but all 24 others were initiated on ART. Major declines in BSIs, and NTS, have been reported to coincide with ART scale-up in both Uganda and Malawi [39], [40]. Another Uganda study [41] found septicemia less common in patients on ART especially after one year on ART. Although still high, the mortality we observed among patients with BSI is likely to have been mitigated by the availability of ART.

A study of BSIs in Blantyre, Malawi [42] found that mycobacterial and pneumococcal bacteremia and sepsis were usually clinically obvious, but that NTS was not usually diagnosed before blood culture results due to the nonspecific clinical signs. Blood cultures are often not feasible in resource limited settings so algorithms for empiric antimicrobial treatment need urgent evaluation.

### Limitations

Despite active tracing of patients the outcomes of 94 (20%) of 469 patients transferred out or lost to follow up are unknown. Some of these patients have probably died [43] which would increase the observed death rates. We were unable to capture timing of loss to follow up as these data were taken from routine programme records. Although, our risk factor analysis was modeled to address the immortal survival bias, the results of this time bias may overstate the beneficial effects of ART in this prospective cohort study [32]. We did not assess the role played by viral load as a determinant of mortality in this study, as access to viral load testing was not available and is not routinely available in clinical practice in Malawi.

### Conclusion

Prompt initiation of ART is vital: more than half of all deaths were among patients who never started ART, most dying within one month of enrollment. ART needs to be seen as an urgent, life-saving, treatment in patients with advanced HIV.

In this patient population intensified laboratory methods for TB diagnosis may offer only modest gains in terms of mortality. In the absence of practically available rapid diagnostics for TB or BSI in resource-poor countries such Malawi, early empiric antimicrobial therapy for TB and bloodstream infections such as NTS needs further evaluation in clinical trials enrolling similar patients.
